# Metal-dependent gene regulation in the causative agent of Lyme disease

**DOI:** 10.3389/fcimb.2013.00079

**Published:** 2013-11-15

**Authors:** Bryan Troxell, X. Frank Yang

**Affiliations:** Department of Immunology and Microbiology, Indiana University School of MedicineIndianapolis, IN, USA

**Keywords:** *Borrelia burgdorferi*, Lyme disease, copper, manganese, zinc, calprotectin

## Abstract

*Borrelia burgdorferi* (*Bb*) is the causative agent of Lyme disease transmitted to humans by ticks of the *Ixodes* spp. *Bb* is a unique bacterial pathogen because it does not require iron (Fe^2+^) for its metabolism. *Bb* encodes a ferritin-like Dps homolog called NapA (also called BicA), which can bind Fe or copper (Cu^2+^), and a manganese (Mn^2+^) transport protein, *Borrelia* metal transporter A (BmtA); both proteins are required for colonization of the tick vector, but BmtA is also required for the murine host. This demonstrates that *Bb*'s metal homeostasis is a critical facet of the complex enzootic life cycle between the arthropod and murine hosts. Although metals are known to influence the expression of virulence determinants during infection, it is unknown how or if metals regulate virulence in *Bb*. Recent evidence demonstrates that *Bb* modulates the intracellular Mn^2+^ and zinc (Zn^2+^) content and, in turn, these metals regulate gene expression through influencing the Ferric Uptake Regulator (Fur) homolog *Borrelia* Oxidative Stress Regulator (BosR). This mini-review focuses on the burgeoning study of metal-dependent gene regulation within *Bb*.

## Introduction

**Figure 1:**
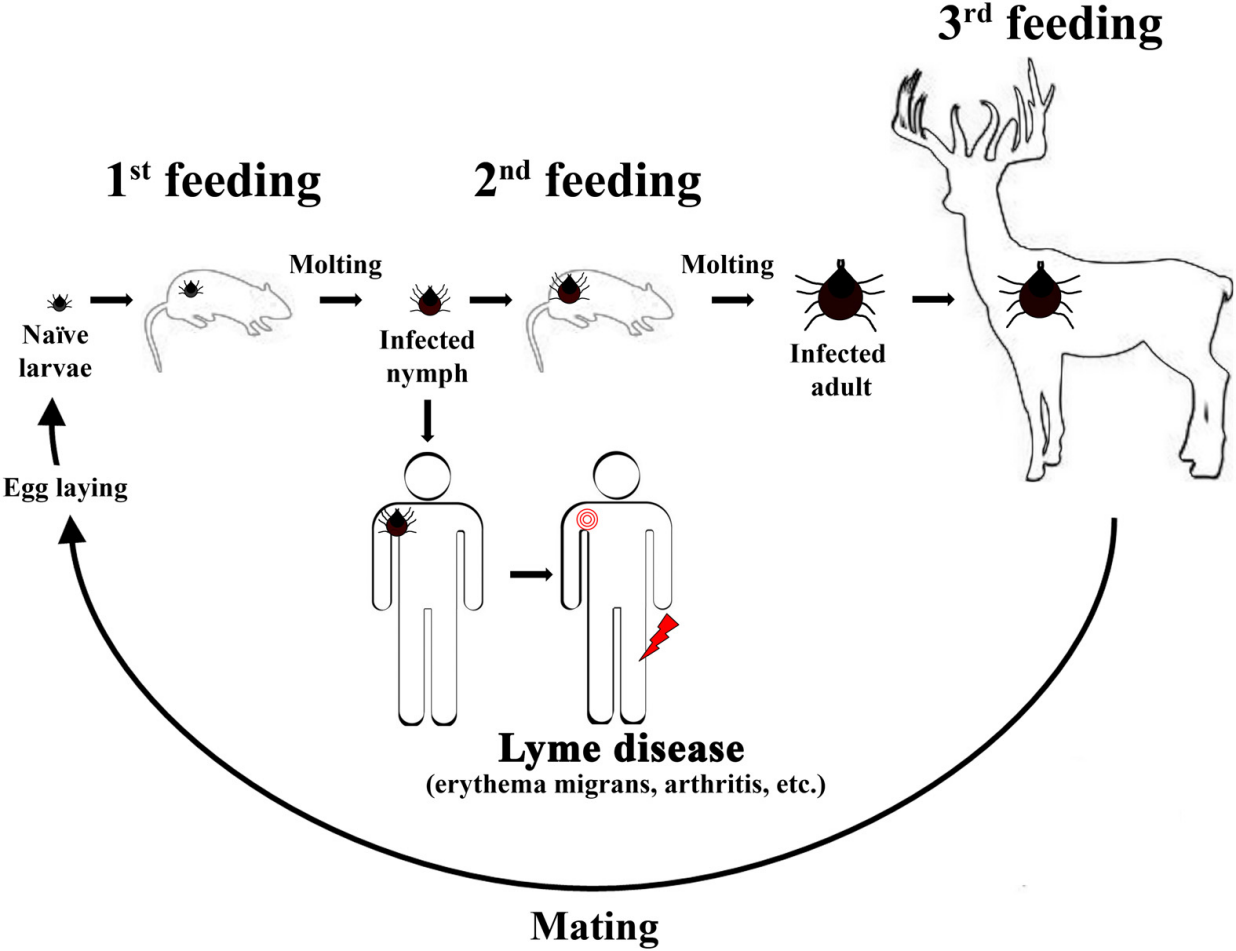
**The usual 2-year enzootic cycle of the Lyme disease spirochete**. A naïve *Ixodes scapularis* larvae will feed on a small rodent near the end of the Summer season or early Fall. The feeding larvae can acquire *Bb* at this feeding (1st feeding) and remain colonized throughout the molting process, which occurs during the Winter season. For the 2nd feeding, infected nymphs will feed late in the Spring season or early in the Summer season. The infected nymphs transmit *Bb* to either a small rodent host, which maintains the enzootic cycle in nature, or humans (accidental host). Infected humans develop Lyme disease and may develop erythema migrans (signified by a red bulls eye near the shoulder in the figure shown) shortly after an infected nymph feeds. Typically, if subject to late Lyme manifestations Lyme disease patients develop Lyme arthritis at one or both knee joints (signified by a red lightning bolt near the knee in the figure shown). For the final feeding (3rd feeding), nymphs will molt and emerge as adults to feed on large mammals, such as deer, during the Fall season. Deer are considered incompetent hosts for *Bb*, but the 3rd feeding is important in the enzootic cycle because female ticks will mate and lay eggs over the Winter season. Naïve larvae will emerge following hatching and the cycle begins anew.

*Borrelia burgdorferi* (*Bb*) is the causative agent of a multisystem disorder known as Lyme disease. *Bb* persists within an enzootic cycle that includes two diverse hosts, a tick vector and a warm-blooded host, typically small rodents. “Hard ticks” of the *Ixodes* genus are important arthropod hosts for colonization by *Bb*. *Ixodes* ticks are slow-feeding ticks (≈48 h for a bloodmeal) that have a 2-year life cycle including three distinct stages: larvae, nymph, and adult (Figure 1). At each stage, ticks will feed once on a warm-blooded host then undergo a molting process, which precedes a period of dormancy that may last months (Figure 1). Because *Bb* colonization of ticks does not appear to occur through transovarial transmittance, unfed larvae ticks are naïve and acquire *Bb* during feeding on an infected warm-blooded host. Feeding ticks can acquire *Bb* at any stage of the usual 2-year life cycle and transmission of *Bb* can occur during feeding on an animal host at any subsequent stage of the life cycle. Small rodents (especially the white-footed mouse, *Peromyscus leucopus*) are the primary animal reservoirs for *Bb* within this enzootic cycle and are sources for the bloodmeal during the larval and nymphal stages (Figure 1). Unlike most bacterial pathogens, *Bb* lacks lipopolysaccharide (LPS), lipooligosaccharide (LOS), and capsule (Radolf and Samuels, 2010). *Bb* is highly motile due to the presence of flagella; however, *Bb*'s flagella are contained within the periplasmic space between the outer and inner membranes. Therefore, *Bb*'s flagella is not surface exposed and is called an endoflagella. The endoflagella are anchored at each end of the cell and provide *Bb* with a characteristic corkscrew movement. Despite *Bb*'s limited metabolism and fastidious nature *Bb* survives within two hosts, a tick vector and a small rodent host. Other animals, such as humans, are infected by *Bb*, but are not considered important for persistence of *Bb* within the enzootic cycle. Of significant interest, *Bb* is one of the few pathogens that does not require iron (Fe^2+^) to grow (Posey and Gherardini, 2000). Given the importance of Fe^2+^ in the regulation of virulence within other bacteria, it is not clear which metals *Bb* utilize for regulating virulence factors. Recent work suggests that metals may play an important role in regulation of virulence within *Bb*.

Metal homeostasis is important to maintain the metabolism of bacterial pathogens. This is accomplished through the combined action of metal transporters, both importers and exporters, which control the abundance of specific metals and the ratio of the transition metals within the cell. Although some metal transporters are highly specific for a cognate metal, others are capable of importing several metals with different affinity of each metal. In addition to the importance of metals in bacterial physiology, metals play a critical role in the control of gene regulation within pathogens. The role of metals within *Bb* is not fully understood. Only a single protein, *Borrelia* metal transporter A (BmtA) is known to participate in metal transport. Analysis of the intracellular metal content with *in vitro* grown *Bb* suggests that BmtA transports Mn^2+^ since this metal is nearly undetectable in ΔbmtA strains (Ouyang et al., 2009a; Troxell et al., 2013). BmtA may also be involved in the import/export of other metals since deletion of *bmtA* alters the intracellular concentrations of Fe^2+^, Cu^2+^, and Zn^2+^ (Wang et al., 2012). The mechanism of BmtA-dependent metal transport is still unknown, but recent evidence indicates that BmtA and Mn^2+^ are involved in regulation of virulence through a Ferric uptake regulator (Fur) homolog named *Borrelia* Oxidative Stress Regulator (BosR). BosR is redox sensing DNA binding protein that utilizes Zn^2+^ as a cofactor (Boylan et al., 2003; Katona et al., 2004). Discussed here is the role of metals in *Bb* physiology and gene expression as it relates to virulence factors required *in vivo*.

## *Bb*: a non-combatant in the war for Fe^2+^

Just as a siege limits the influx of food and supplies to an enemy's stronghold, during infection the host transports metals away from the locale of pathogens and synthesizes copious amounts of metal-chelating proteins to limit access of these essential micronutrients. The hosts' ability to produce metal-chelating proteins is important for defending against pathogens since deletion of the chelating protein calprotectin enhances virulence of *Acinetobacter baumannii*, *Staphylococcus aureus*, and the opportunistic yeast pathogen *Candida albicans* (Corbin et al., 2008; Kehl-Fie et al., 2011; Damo et al., 2013). Calprotectin can bind Mn^2+^ and Zn^2+^ and is an abundant protein present in neutrophils (Yui et al., 2003), which are an early host defender against invading pathogens. Some bacterial pathogens are capable of overcoming the growth inhibition exerted by calprotectin; *Salmonella enterica* serovar Typhimurium (*S*. Typhimurium) expresses a high affinity Zn^2+^ ATP-binding cassette (ABC) transport system that outcompetes Zn^2+^ chelation by calprotectin (Liu et al., 2012). Calprotectin is known to inhibit *in vitro* growth of *Bb* through Zn^2+^ sequestration (Lusitani et al., 2003). The contribution of calprotectin to *Bb* growth *in vivo* is unknown, but *Bb* encodes several putative uncharacterized ABC transporters that could be involved in metal transport during infection. In addition, whether calprotectin inhibits *Bb* growth through Mn^2+^ chelation is unknown. The fierce war between the pathogen and host for accessibility of Fe^2+^ poses a problem to pathogens; however, *Bb* has evolved a novel solution by becoming a non-combatant in the war for Fe^2+^. *Bb* does not appear to transport Fe^2+^, lacks many biosynthetic and catabolic pathways that require Fe^2+^, and exhibits no defect in growth in the absence of detectable Fe^2+^ (Posey and Gherardini, 2000). Although a recent study indicates there is detectable Fe^2+^ within *Bb*, the physiological relevance of this finding remains uncertain (Wang et al., 2012). Another study did not detect intracellular Fe^2+^ following *in vitro* cultivation of *Bb* (Aguirre et al., 2013). Therefore, additional experiments are required to address these discrepancies. At this point, how Fe^2+^ is transported within *Bb* is unknown. Future work is required to determine the contribution of intracellular Fe^2+^ to *Bb* gene regulation and metabolism. Instead, because calprotectin inhibits *Bb* growth by Zn^2+^ sequestration, the existing data suggests that Zn^2+^ is an important metal within the metabolism of *Bb*. This is supported by the Zn^2+^-dependent enzymatic activity of peptide deformylase (Nguyen et al., 2007) and the glycolytic enzyme fructose-1,6-bisphosphate aldolase (Bourret et al., 2011). Furthermore, peptide deformylase may be an essential enzyme (Jain et al., 2005) and, since glycolysis is the sole mechanism for the generation of ATP within *Bb*, Zn^2+^ may be a critical metal for *Bb*.

## BicA and BmtA: two proteins with novel function within *Bb*

Bacteria encode metal binding proteins (ferritins or ferritin-like proteins) that store metals and serve as a facile source of essential metals when encountering a metal-depleted environment. *Bb* encodes a metal binding protein (NapA or BicA) that exhibits homology to the ferritin-like Dps present in other bacteria. Purified BicA is capable of binding Fe^2+^ or Cu^2+^, but lacks either metal when isolated (Li et al., 2007; Wang et al., 2012). The majority of studies have focused on the role of Fe^2+^ chelation by the host in nutritional immunity, but recent evidence demonstrates the importance of chelating Zn^2+^ and Mn^2+^ in thwarting bacterial infections (Kehl-Fie and Skaar, 2010). However, as part of the antimicrobial defense present within ticks, an antimicrobial peptide, known as microplusin, inhibits bacterial growth by Cu^2+^ chelation (Silva et al., 2009, 2011). Microplusin is expressed within the hemocele of ticks (Esteves et al., 2009), implying that this locale is a Cu^2+^ limited environment. *Bb* does appear to regulate its intracellular Cu^2+^, but the relevance or need for Cu^2+^ is unknown (Wang et al., 2012). The importance of BicA to the enzootic cycle is restricted to residence within the tick vector (Li et al., 2007), implying that Zn^2+^ and Cu^2+^ are limiting within this host.

The role of Mn^2+^ in *Bb* metabolism is not understood. The gene *bmtA*, encoding a Mn^2+^ transport protein BmtA, is not essential for *in vitro* growth within virulent *Bb* strains from the B31 (tick isolated) and 297 (human isolated) lineages despite reducing cellular Mn^2+^ to near undetectable concentrations (Troxell et al., 2013). *Bb* cultivation *in vitro* requires a complex growth medium called BSK (Barbour, 1984). Treatment of BSK medium with a chelating resin, called Chelex, results in significant changes of the concentrations of metals. Chelex treatment of BSK reduces Zn^2+^, but Mn^2+^ becomes undetectable in the medium. Despite the undetectable Mn^2+^ in Chelex-treated BSK growth medium, no growth defects are observed for wild-type or Δ*bmtA* strains during cultivation in this medium (Troxell et al., 2013). BmtA has homology to the GufA family of metal transporters (Guerinot, 2000). BmtA has 8 membrane spanning domains and is predicted to transport cations through a novel mechanism (Ouyang et al., 2009a). To date, only a single protein within *Bb* is characterized as being Mn^2+^-dependent; specifically, the superoxide dismutase (SOD) encoded by *sodA* (Troxell et al., 2012; Aguirre et al., 2013). The expression of *bmtA* and the intracellular concentration of Mn^2+^ are enhanced during cultivation at 25°C, suggesting there may be a requirement for Mn^2+^ at cooler temperatures (Ojaimi et al., 2003; Troxell et al., 2013). The physiological need for more Mn^2+^ at 25°C is unknown, but this may be due to the need for defense against reactive oxygen species (ROS) because *Bb* encodes a Mn-dependent SOD and lower temperatures contain increased concentrations of dissolved O_2_ that could lead to enhanced formation of superoxide radical (O^−^_2_) (Troxell et al., 2012; Aguirre et al., 2013). However, *Bb* may encode additional proteins that require Mn^2+^.

Mn^2+^ is considered an essential trace element within biology. In bacteria, Mn^2+^ is critical for defense against several stresses such as oxidative stress, bile stress, and resistance to antibiotics (Anjem et al., 2009; Srinivasan et al., 2012). In addition, Mn^2+^ is involved in gene regulation through indirect mechanisms. For instance, the alarmone guanosine tetraphosphate (ppGpp) is synthesized and degraded by SpoT/RelA homolog proteins. During conditions of nutrient deprivation, ppGpp is synthesized and binds to the RNA polymerase (RNAP) in order to enhance transcription of genes important for survival or virulence while reducing transcription of genes involved in growth and cell division (Magnusson et al., 2005). SpoT/RelA homologs contain a highly conserved Mn^2+^ binding site and require Mn^2+^ as a cofactor for the enzymatic degradation of ppGpp (Sy, 1977; Sun et al., 2010). *Bb* encodes a SpoT/RelA homolog, *bb0198*, that is induced during serum starvation and is responsible for both synthesis and degradation of ppGpp (Concepcion and Nelson, 2003; Bugrysheva et al., 2005). This suggests that *Bb* may require Mn^2+^ in order to initiate cell growth. Recently, *Bb*'s peptide deformylase was isolated with bound Mn^2+^ (Aguirre et al., 2013); however, an enzymatic assay of the Mn-bound enzyme was not conducted. Whether peptide deformylase functions with Mn^2+^ is unknown, but this enzyme is active with Zn^2+^ as a cofactor (Nguyen et al., 2007). Future work is needed to determine the metal specificity of BB0198 and the peptide deformylase and to identify *Bb* proteins that require Mn^2+^.

Surprisingly, some enhancement in the intracellular concentration of Zn^2+^ for Δ*bmtA* has been noted (Wang et al., 2012). It has been hypothesized that within Δ*bmtA* there may be compensation for the reduction of Mn^2+^ by enhancing the transport of Zn^2+^ and thereby replacing the requirement of Mn^2+^ with Zn^2+^. Although future work is required to fully test this hypothesis, the replacement of Mn^2+^ for Zn^2+^ in Mn^2+^-dependent enzymes causes a pronounced reduction in catalytic efficiency or abrogates enzymatic activity altogether (Ose and Fridovich, 1976; Sobota and Imlay, 2011; Gu and Imlay, 2013). Metal-dependent transcription factors can utilize a variety of metals for function, i.e., Mn^2+^ or Fe^2+^ in the case of Fur (Privalle and Fridovich, 1993), and host metal-sequestering proteins exhibit promiscuity in metal binding, which is demonstrated by the Mn^2+^ or Zn^2+^ binding site (S1 site) in calprotectin (Damo et al., 2013). This is in contrast to metal-dependent enzymes, which exhibit stringent metal specificity for activity, as is the case for SpoT/RelA homologs and *Bb*'s SodA (Sy, 1977; Troxell et al., 2012; Aguirre et al., 2013). However, because many of *Bb*'s putative metalloenzymes are uncharacterized, the possibility exists that a significant number of these proteins can utilize either Mn^2+^ or Zn^2+^ within the cell.

## *Bb*'s metal requirement within the tick

The unfed tick is presumed to be a nutrient deprived environment for *Bb*. Starvation conditions may mimic oxidative stress conditions and factors responsible for defense against ROS are also important for survival during starvation (Jenkins et al., 1988; Nystrom et al., 1996). *Bb* may require Mn^2+^ in order to defend against ROS that occurs during onset of the bloodmeal. Although Mn^2+^ complexed with other biological compounds, such as bicarbonate, are capable of degrading ROS, this requires large concentrations of intracellular Mn^2+^ that occurs within *Lactobacillus plantarum* (Archibald and Fridovich, 1981, 1982; Stadtman et al., 1990). In the only report to compare directly the intracellular Mn^2+^ content of *L. plantarum* with *Bb*, it was observed that *Bb* contains 20 to 100-fold lower intracellular Mn^2+^ compared to *L. plantarum*, indicating this is an unlikely mechanism for ROS defense within *Bb* (Posey and Gherardini, 2000). However, *Bb*'s intracellular Mn^2+^ can fluctuate during *in vitro* growth conditions (Troxell et al., 2013), suggesting that environmental conditions within the tick-mouse life cycle may exist whereby *Bb* could contain sufficient intracellular Mn^2+^ to degrade ROS in a manner similar to *L. plantarum*. Although *sodA* is required for infection of the murine host (Esteve-Gassent et al., 2009), the contribution of *sodA* within the tick vector is unknown. It is currently unclear if *Bb* contains a high intracellular Mn^2+^ within the unfed tick or is starved for metals. Because of the involvement of BicA in Cu^2+^ and Zn^2+^ homeostasis and since Δ*bicA* exhibits a defect within the unfed tick (Li et al., 2007), the results support the notion that these two metals are limiting. In addition, the contribution of BmtA to the unfed tick is unknown.

## Regulation of σ^S^ by Zn^2+^ and Mn^2+^ within *Bb*

*Bb* is capable of surviving within two diverse hosts through changes in gene expression, specifically outer surface lipoproteins that modulate adaptation within each host. Outer Surface Proteins A (OspA) and C (OspC) are a lipoproteins produced by *Bb* within the tick and animal host, respectively. *Bb* contains a limited genome that contains a relatively small number of transcription factors and sigma factors: *Bb* encodes only three sigma factors the housekeeping σ^70^, and two alternative sigma factors, RpoN (σ^54^) and RpoS (σ^S^) (Fraser et al., 1997; Samuels, 2011; Radolf et al., 2012). In addition, *Bb* genome encodes only one bacterial enhancer binding protein (bEBP), known as Rrp2, which is involved in σ^54^ activation. The requirement of the Rrp2-RpoN-RpoS pathway (or Rrp2-σ^54^−σ^S^ sigma factor cascade) in the regulation of *ospA* and *ospC* demonstrates the importance of this regulatory network (Hubner et al., 2001; Yang et al., 2003; Caimano et al., 2004; Fisher et al., 2005; Gilbert et al., 2007). Rrp2 and σ^54^ directly activates transcription of *rpoS* (Smith et al., 2007; Blevins et al., 2009). σ^S^ then activates transcription of *ospC* by direct binding to the promoter of *ospC* (Yang et al., 2005) and also represses expression of *ospA* (Caimano et al., 2007). In addition, BosR, a Fur/PerR-like family transcription factor and a Zn^2+^-dependent DNA binding protein, has been shown to be essential for transcription of *rpoS* (Ouyang et al., 2009b, 2011). More recently, Wang et al. demonstrated that BosR may also directly repress *ospA* (Wang et al., 2013). Because RpoS regulates many genes important for *Bb* transmission and mammalian infection such as *ospC*, this pathway is essential for the enzootic cycle of *Bb* (Caimano et al., 2004; Grimm et al., 2004; Pal et al., 2004; Boardman et al., 2008; Ouyang et al., 2008). Moreover, *bosR* is required for transmission from the tick vector and infection of the mammalian host (Hyde et al., 2009; Ouyang et al., 2009b). Thus, *Bb* has evolved to utilize the transcription factor BosR for virulence.

*Bb* is a highly fastidious pathogen. The cultivation of *Bb* requires a complex medium that is analogous to cell culture media for eukaryotic cells (Barbour, 1984). Comparisons of *Bb* replication within a feeding tick and during *in vitro* growth at 35–37°C demonstrate that both conditions support growth with a generation time of ≈8–10 h (De Silva and Fikrig, 1995). Metal analysis of the cultivation medium for *Bb* indicates there is ≈5 μ M Zn^2+^, ≈4 μM Cu^2+^, and ≈0.1 μM Mn^2+^ (Wang et al., 2012; Troxell et al., 2013). Besides Fe^2+^, other transition metals, such as Zn^2+^ and Mn^2+^ are known to influence gene regulation within bacterial pathogens (Corbin et al., 2008). Based on the Zn^2+^-dependent nature of BosR (Boylan et al., 2003; Katona et al., 2004), and because BosR regulates *rpoS*, Zn^2+^ could regulate *rpoS* within *Bb*.

Metal analysis indicates that while intracellular Zn^2+^ remained relatively constant under different conditions, Mn^2+^ was subject to temperature-dependent regulation within *Bb* (Troxell et al., 2013). Moreover, the intracellular Mn^2+^ can fluctuate 20-fold during *in vitro* growth conditions and the temperature-dependent inverse concentration of intracellular of Mn^2+^ is reminiscent of the inverse regulation of *ospA* and *ospC* within *Bb* (Stevenson et al., 1995; Obonyo et al., 1999; Yang et al., 2000; Alverson et al., 2003). To test if Mn^2+^ could suppress regulation by σ^S^, MnCl_2_ was added to cultures growing under conditions of σ^S^ activation. The addition of MnCl_2_ increases intracellular Mn^2+^ and reduces the expression of *rpoS* and σ^S^-activated *ospC* (Troxell et al., 2013). The addition of excess ZnSO_4_ increases the intracellular Zn^2+^, increases the level of BosR protein, and abrogates the repression of *rpoS* by Mn^2+^. Surprisingly, MnCl_2_ did not influence transcription of *bosR*, but reduced the level of the BosR protein (Troxell et al., 2013). In addition, deletion of *bmtA* in two infectious strains does not alter *bosR* transcription, but enhances temperature-dependent activation in the level of BosR, which results in increased transcription of *rpoS* and *ospC*. As an earlier study shows, the BosR protein level is increased by CO_2_ despite the inability of dissolved CO_2_ to regulate transcription of *bosR* (Hyde et al., 2007). These combined results suggest that either metals or CO_2_ may control the level of the BosR protein, which activates transcription of *rpoS*. Correlation of the intracellular Mn^2+^:Zn^2+^ indicates that the ratio between these two metals play an important role in the level of BosR protein and *rpoS* regulation. Collectively, these results support the hypothesis that a combined reduction in intracellular Mn^2+^ while increasing Zn^2+^ regulates σ^S^ by dramatically enhancing the level of BosR protein.

**Figure 2:**
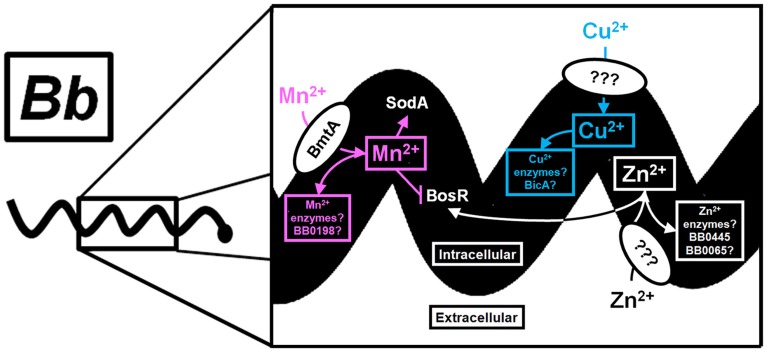
**Known and putative roles of Mn^2+^, Cu^2+^, and Zn^2+^ in gene regulation and metabolism of *Bb***. A schematic of the importance of transition metals within *Bb* is shown with a magnification of a section from a single *Bb* cell. Extracellular Mn^2+^ is transported through BmtA and supplies the appropriate cofactor for the Mn-SOD and possibly the SpoT/RelA homolog BB0198 (designated by a pink arrow). In addition, Mn^2+^ reduces the level of BosR protein (designated by a pink blunted line), which controls transcription of the alternative sigma factor, *rpoS* (not shown). The putative role of Mn^2+^ as a cofactor for additional unknown enzymes is shown with a pink box. Zn^2+^ transport is uncharacterized in *Bb*, but is presumed to be transported by a membrane bound protein. The requirement for Zn^2+^ within *Bb* is likely to include enzymes within glycolysis, such as fructose 1,6-bisphosphatase (BB0445), and the peptide deformylase (BB0065) shown in the white box. Zn^2+^ is a known cofactor for the DNA binding protein BosR. Therefore, the intracellular Mn^2+^:Zn^2+^ can modulate the level of BosR protein. The transporter for Cu^2+^ and the role of Cu^2+^ within *Bb* is unknown, but BicA may be involved in transport and homeostasis (blue box). Moreover, the contribution of Cu^2+^ to gene regulation within *Bb* is unknown, but is predicted to involve redox sensing transcription factors (Changela et al., 2003; Gomez-Santos et al., 2011). Future work is required to elucidate the complete role of these metals in gene regulation and physiology of this important vector borne pathogen.

Why does *Bb* require *bmtA* for the enzootic cycle? The presence of excess Mn^2+^ suggests there is a collection of unknown targets that require Mn^2+^ for activity. One function may be to control σ^S^ activation during the enzootic cycle. Precise regulation of *ospC* and other outer surface proteins, such as *vlsE*, is required for infection of the murine host; constitutive activation of either surface protein results in rapid elimination of *Bb* by either innate cells or the humoral response of the host (Liang et al., 2002; Xu et al., 2006, 2008a,b). In the absence of any defined metabolic requirement for Mn^2+^ within *Bb*, the importance of Mn^2+^ to the enzootic cycle could be to control regulation of highly immunogenic outer surface proteins. Nevertheless, the limited genome of *Bb* encodes several homologs that may require Mn^2+^ for activity. Furthermore, BmtA appears to influence not only the intracellular Mn^2+^ concentration, but also the concentrations of Cu^2+^ and Zn^2+^. How Zn^2+^ and Cu^2+^ are transported within *Bb* is unknown, but it is likely that these metals are required for proper regulation of virulence genes and unknown metabolic genes. Future work will no doubt shed light on the importance of these metals as cofactors and their influence on gene regulation. This is summarized in Figure 2, which depicts the known and putative roles of Mn^2+^, Cu^2+^, and Zn^2+^ within *Bb*.

## Conclusions

Unlike most bacterial pathogens, *Bb* does not require Fe^2+^ for growth, which presents a unique model system to study metal-dependent gene regulation and stress responses. The bloodmeal is rich in Zn^2+^/Cu^2+^ and relatively poor in Mn^2+^, which suggests that *Bb*'s intracellular Zn^2+^/Cu^2+^ content may increase through unidentified transporters (Figure 2). Because Mn^2+^ regulates the BosR protein level, but not *bosR* transcription (Troxell et al., 2013), the low Mn^2+^ content in blood may further enhance expression *rpoS*, which is required for *Bb* to exit the tick midgut and reach the salivary glands during tick feeding (Fisher et al., 2005; Dunham-Ems et al., 2012). How does *Bb* coordinate the regulation of transport of Mn^2+^, Cu^2+^, and Zn^2+^ during the enzootic cycle? What is apparent from *in vitro* work is that the intracellular Mn^2+^:Zn^2+^ ratio regulates transcription of the alternative sigma factor, *rpoS*, which controls activation of genes required for infection of mammals. A caveat to these studies is the heavy reliance on *in vitro* experiments due to the difficulties of measuring intracellular metal content while detecting changes in gene expression during *in vivo* studies. Infection studies with Δ*bicA* and Δ*bmtA* demonstrate the importance of these genes within the enzootic cycle, but the mechanism for why *Bb* requires them is unknown. Although the contribution to the unfed tick is known for *bicA*, the contribution of *bmtA* to survival within the dormant tick is unknown. How BicA and BmtA control metal homeostasis or gene expression *in vivo* would greatly improve our understanding of their importance in infection. Moreover, the identification of a dedicated Zn transport system and Cu transport system within *Bb* would provide additional and much needed clarity. It is clear that we are only beginning to understand the importance of metals in the metabolism and gene regulation within the Lyme disease spirochete

### Conflict of interest statement

The authors declare that the research was conducted in the absence of any commercial or financial relationships that could be construed as a potential conflict of interest.

## References

[B1] Aguirre J. D. Clark H. M. McIlvin M. Vazquez C. Palmere S. L. Grab D. J. (2013). A manganese-rich environment supports superoxide dismutase activity in a Lyme disease pathogen, *Borrelia burgdorferi*. J. Biol. Chem. 288, 8468–8478 10.1074/jbc.M112.433540 23376276 PMC3605662

[B2] Alverson J. Bundle S. F. Sohaskey C. D. Lybecker M. C. Samuels D. S. (2003). Transcriptional regulation of the ospAB and ospC promoters from *Borrelia burgdorferi*. Mol. Microbiol. 48, 1665–1677 10.1046/j.1365-2958.2003.03537.x 12791146

[B3] Anjem A. Varghese S. Imlay J. A. (2009). Manganese import is a key element of the OxyR response to hydrogen peroxide in *Escherichia coli*. Mol. Microbiol. 72, 844–858 10.1111/j.1365-2958.2009.06699.x 19400769 PMC2776087

[B4] Archibald F. S. Fridovich I. (1981). Manganese and defenses against oxygen toxicity in *Lactobacillus plantarum*. J. Bacteriol. 145, 442–451 6257639 10.1128/jb.145.1.442-451.1981 PMC217292

[B5] Archibald F. S. Fridovich I. (1982). The scavenging of superoxide radical by manganous complexes: *in vitro*. Arch. Biochem. Biophys. 214, 452–463 10.1016/0003-9861(82)90049-2 6284026

[B6] Barbour A. G. (1984). Isolation and cultivation of Lyme disease spirochetes. Yale J. Biol. Med. 57, 521–525 6393604 PMC2589996

[B7] Blevins J. S. Xu H. He M. Norgard M. V. Reitzer L. Yang X. F. (2009). Rrp2, a sigma54-dependent transcriptional activator of *Borrelia burgdorferi*, activates rpoS in an enhancer-independent manner. J. Bacteriol. 191, 2902–2905 10.1128/JB.01721-08 19201806 PMC2668385

[B8] Boardman B. K. He M. Ouyang Z. Xu H. Pang X. Yang X. F. (2008). Essential role of the response regulator Rrp2 in the infectious cycle of *Borrelia burgdorferi*. Infect. Immun. 76, 3844–3853 10.1128/IAI.00467-08 18573895 PMC2519420

[B9] Bourret T. J. Boylan J. A. Lawrence K. A. Gherardini F. C. (2011). Nitrosative damage to free and zinc-bound cysteine thiols underlies nitric oxide toxicity in wild-type *Borrelia burgdorferi*. Mol. Microbiol. 81, 259–273 10.1111/j.1365-2958.2011.07691.x 21564333 PMC3147059

[B10] Boylan J. A. Posey J. E. Gherardini F. C. (2003). Borrelia oxidative stress response regulator, BosR: a distinctive Zn-dependent transcriptional activator. Proc. Natl. Acad. Sci. U.S.A. 100, 11684–11689 10.1073/pnas.2032956100 12975527 PMC208818

[B11] Bugrysheva J. V. Bryksin A. V. Godfrey H. P. Cabello F. C. (2005). *Borrelia burgdorferi* rel is responsible for generation of guanosine-3'-diphosphate-5'-triphosphate and growth control. Infect. Immun. 73, 4972–4981 10.1128/IAI.73.8.4972-4981.2005 16041012 PMC1201186

[B12] Caimano M. J. Eggers C. H. Hazlett K. R. Radolf J. D. (2004). RpoS is not central to the general stress response in *Borrelia burgdorferi* but does control expression of one or more essential virulence determinants. Infect. Immun. 72, 6433–6445 10.1128/IAI.72.11.6433-6445.2004 15501774 PMC523033

[B12a] Caimano M. J. Iyer R. Eggers C. H. Gonzalez C. Morton E. A. Gilbert M. A. (2007). Analysis of the RpoS regulon in *Borrelia burgdorferi* in response to mammalian host signals provides insight into RpoS function during the enzootic cycle. Mol. Microbiol. 65, 1193–1217 10.1111/j.1365-2958.2007.05860.x 17645733 PMC2967192

[B13] Changela A. Chen K. Xue Y. Holschen J. Outten C. E. O'Halloran T. V. (2003). Molecular basis of metal-ion selectivity and zeptomolar sensitivity by CueR. Science 301, 1383–1387 10.1126/science.1085950 12958362

[B14] Concepcion M. B. Nelson D. R. (2003). Expression of spoT in *Borrelia burgdorferi* during serum starvation. J. Bacteriol. 185, 444–452 10.1128/JB.185.2.444-452.2003 12511489 PMC145309

[B15] Corbin B. D. Seeley E. H. Raab A. Feldmann J. Miller M. R. Torres V. J. (2008). Metal chelation and inhibition of bacterial growth in tissue abscesses. Science 319, 962–965 10.1126/science.1152449 18276893

[B16] Damo S. M. Kehl-Fie T. E. Sugitani N. Holt M. E. Rathi S. Murphy W. J. (2013). Molecular basis for manganese sequestration by calprotectin and roles in the innate immune response to invading bacterial pathogens. Proc. Natl. Acad. Sci. U.S.A. 110, 3841–3846 10.1073/pnas.1220341110 23431180 PMC3593839

[B17] De Silva A. M. Fikrig E. (1995). Growth and migration of *Borrelia burgdorferi* in Ixodes ticks during blood feeding. Am. J. Trop. Med. Hyg. 53, 397–404 7485694 10.4269/ajtmh.1995.53.397

[B18] Dunham-Ems S. M. Caimano M. J. Eggers C. H. Radolf J. D. (2012). *Borrelia burgdorferi* requires the alternative sigma factor RpoS for dissemination within the vector during tick-to-mammal transmission. PLoS Pathog. 8:e1002532 10.1371/journal.ppat.1002532 22359504 PMC3280991

[B19] Esteve-Gassent M. D. Elliott N. L. Seshu J. (2009). sodA is essential for virulence of *Borrelia burgdorferi* in the murine model of Lyme disease. Mol. Microbiol. 71, 594–612 10.1111/j.1365-2958.2008.06549.x 19040638

[B20] Esteves E. Fogaca A. C. Maldonado R. Silva F. D. Manso P. P. Pelajo-Machado M. (2009). Antimicrobial activity in the tick *Rhipicephalus (Boophilus) microplus* eggs: cellular localization and temporal expression of microplusin during oogenesis and embryogenesis. Dev. Comp. Immunol. 33, 913–919 10.1016/j.dci.2009.02.009 19454333

[B21] Fisher M. A. Grimm D. Henion A. K. Elias A. F. Stewart P. E. Rosa P. A. (2005). Borrelia burgdorferi sigma54 is required for mammalian infection and vector transmission but not for tick colonization. Proc. Natl. Acad. Sci. U.S.A. 102, 5162–5167 10.1073/pnas.0408536102 15743918 PMC555983

[B22] Fraser C. M. Casjens S. Huang W. M. Sutton G. G. Clayton R. Lathigra R. (1997). Genomic sequence of a Lyme disease spirochaete, *Borrelia burgdorferi*. Nature 390, 580–586 10.1038/37551 9403685

[B23] Gilbert M. A. Morton E. A. Bundle S. F. Samuels D. S. (2007). Artificial regulation of *ospC* expression in *Borrelia burgdorferi*. Mol. Microbiol. 63, 1259–1273 10.1111/j.1365-2958.2007.05593.x 17257307

[B24] Gomez-Santos N. Perez J. Sanchez-Sutil M. C. Moraleda-Munoz A. Munoz-Dorado J. (2011). CorE from *Myxococcus xanthus* is a copper-dependent RNA polymerase sigma factor. PLoS Genet. 7:e1002106 10.1371/journal.pgen.1002106 21655090 PMC3107203

[B25] Grimm D. Tilly K. Byram R. Stewart P. E. Krum J. G. Bueschel D. M. (2004). Outer-surface protein C of the Lyme disease spirochete: a protein induced in ticks for infection of mammals. Proc. Natl. Acad. Sci. U.S.A. 101, 3142–3147 10.1073/pnas.0306845101 14970347 PMC365757

[B26] Gu M. Imlay J. A. (2013). Superoxide poisons mononuclear iron enzymes by causing mismetallation. Mol. Microbiol. 89, 123–134 10.1111/mmi.12263 23678969 PMC3731988

[B27] Guerinot M. L. (2000). The ZIP family of metal transporters. Biochim. Biophys. Acta 1465, 190–198 10.1016/S0005-2736(00)00138-3 10748254

[B28] Hubner A. Yang X. Nolen D. M. Popova T. G. Cabello F. C. Norgard M. V. (2001). Expression of *Borrelia burgdorferi* OspC and DbpA is controlled by a RpoN-RpoS regulatory pathway. Proc. Natl. Acad. Sci. U.S.A. 98, 12724–12729 10.1073/pnas.231442498 11675503 PMC60121

[B29] Hyde J. A. Shaw D. K. Smith Iii R. Trzeciakowski J. P. Skare J. T. (2009). The BosR regulatory protein of *Borrelia burgdorferi* interfaces with the RpoS regulatory pathway and modulates both the oxidative stress response and pathogenic properties of the Lyme disease spirochete. Mol. Microbiol. 74, 1344–1355 10.1111/j.1365-2958.2009.06951.x 19906179 PMC2805275

[B30] Hyde J. A. Trzeciakowski J. P. Skare J. T. (2007). *Borrelia burgdorferi* alters its gene expression and antigenic profile in response to CO2 levels. J. Bacteriol. 189, 437–445 10.1128/JB.01109-06 17098904 PMC1797391

[B31] Jain R. Chen D. White R. J. Patel D. V. Yuan Z. (2005). Bacterial Peptide deformylase inhibitors: a new class of antibacterial agents. Curr. Med. Chem. 12, 1607–1621 10.2174/0929867054367194 16022661

[B32] Jenkins D. E. Schultz J. E. Matin A. (1988). Starvation-induced cross protection against heat or H2O2 challenge in *Escherichia coli*. J. Bacteriol. 170, 3910–3914 3045081 10.1128/jb.170.9.3910-3914.1988 PMC211389

[B33] Katona L. I. Tokarz R. Kuhlow C. J. Benach J. Benach J. L. (2004). The fur homologue in *Borrelia burgdorferi*. J. Bacteriol. 186, 6443–6456 10.1128/JB.186.19.6443-6456.2004 15375125 PMC516618

[B34] Kehl-Fie T. E. Chitayat S. Hood M. I. Damo S. Restrepo N. Garcia C. (2011). Nutrient metal sequestration by calprotectin inhibits bacterial superoxide defense, enhancing neutrophil killing of *Staphylococcus aureus*. Cell Host Microbe 10, 158–164 10.1016/j.chom.2011.07.004 21843872 PMC3157011

[B35] Kehl-Fie T. E. Skaar E. P. (2010). Nutritional immunity beyond iron: a role for manganese and zinc. Curr. Opin. Chem. Biol. 14, 218–224 10.1016/j.cbpa.2009.11.008 20015678 PMC2847644

[B36] Li X. Pal U. Ramamoorthi N. Liu X. Desrosiers D. C. Eggers C. H. (2007). The Lyme disease agent *Borrelia burgdorferi* requires BB0690, a Dps homologue, to persist within ticks. Mol. Microbiol. 63, 694–710 10.1111/j.1365-2958.2006.05550.x 17181780

[B37] Liang F. T. Jacobs M. B. Bowers L. C. Philipp M. T. (2002). An immune evasion mechanism for spirochetal persistence in Lyme borreliosis. J. Exp. Med. 195, 415–422 10.1084/jem.20011870 11854355 PMC2193615

[B38] Liu J. Z. Jellbauer S. Poe A. J. Ton V. Pesciaroli M. Kehl-Fie T. E. (2012). Zinc sequestration by the neutrophil protein calprotectin enhances Salmonella growth in the inflamed gut. Cell Host Microbe 11, 227–239 10.1016/j.chom.2012.01.017 22423963 PMC3308348

[B39] Lusitani D. Malawista S. E. Montgomery R. R. (2003). Calprotectin, an abundant cytosolic protein from human polymorphonuclear leukocytes, inhibits the growth of *Borrelia burgdorferi*. Infect. Immun. 71, 4711–4716 10.1128/IAI.71.8.4711-4716.2003 12874352 PMC166021

[B40] Magnusson L. U. Farewell A. Nystrom T. (2005). ppGpp: a global regulator in *Escherichia coli*. Trends Microbiol. 13, 236–242 10.1016/j.tim.2005.03.008 15866041

[B41] Nguyen K. T. Wu J. C. Boylan J. A. Gherardini F. C. Pei D. (2007). Zinc is the metal cofactor of *Borrelia burgdorferi* peptide deformylase. Arch. Biochem. Biophys. 468, 217–225 10.1016/j.abb.2007.09.023 17977509 PMC2151311

[B42] Nystrom T. Larsson C. Gustafsson L. (1996). Bacterial defense against aging: role of the *Escherichia coli* ArcA regulator in gene expression, readjusted energy flux and survival during stasis. EMBO J. 15, 3219–3228 8670822 PMC451874

[B43] Obonyo M. Munderloh U. G. Fingerle V. Wilske B. Kurtti T. J. (1999). *Borrelia burgdorferi* in tick cell culture modulates expression of outer surface proteins A and C in response to temperature. J. Clin. Microbiol. 37, 2137–2141 10364575 10.1128/jcm.37.7.2137-2141.1999 PMC85101

[B44] Ojaimi C. Brooks C. Casjens S. Rosa P. Elias A. Barbour A. (2003). Profiling of temperature-induced changes in *Borrelia burgdorferi* gene expression by using whole genome arrays. Infect. Immun. 71, 1689–1705 10.1128/IAI.71.4.1689-1705.2003 12654782 PMC152086

[B45] Ose D. E. Fridovich I. (1976). Superoxide dismutase. Reversible removal of manganese and its substitution by cobalt, nickel or zinc. J. Biol. Chem. 251, 1217–1218 765340

[B46] Ouyang Z. Blevins J. S. Norgard M. V. (2008). Transcriptional interplay among the regulators Rrp2, RpoN, and RpoS in *Borrelia burgdorferi*. Microbiology 154, 2641–2658 10.1099/mic.0.2008/019992-0 18757798

[B47] Ouyang Z. Deka R. K. Norgard M. V. 2011 Bos R (BB0647)controls the RpoN-RpoS regulatory pathway and virulence expression in *Borrelia burgdorferi* by a novel DNA-binding mechanism. PLoS. Pathog 7, e1001272 10.1371/journal.ppat.1001272 21347346 PMC3037356

[B49a] Ouyang Z. He M. Oman T. Yang X. F. Norgard M. V. (2009a). A manganese transporter, BB0219 (BmtA), is required for virulence by the Lyme disease spirochete, *Borrelia burgdorferi*. Proc. Natl. Acad. Sci. U.S.A. 106, 3449–3454 10.1073/pnas.0812999106 19218460 PMC2651281

[B48] Ouyang Z. Kumar M. Kariu T. Haq S. Goldberg M. Pal U. (2009b). BosR (BB0647) governs virulence expression in *Borrelia burgdorferi*. Mol. Microbiol. 74, 1331–1343 10.1111/j.1365-2958.2009.06945.x 19889086 PMC2831293

[B49] Pal U. Yang X. Chen M. Bockenstedt L. K. Anderson J. F. Flavell R. A. (2004). OspC facilitates *Borrelia burgdorferi* invasion of *Ixodes scapularis* salivary glands. J. Clin. Invest. 113, 220–230 10.1172/JCI19894 14722614 PMC311436

[B50] Posey J. E. Gherardini F. C. (2000). Lack of a role for iron in the Lyme disease pathogen. Science 288, 1651–1653 10.1126/science.288.5471.1651 10834845

[B51] Privalle C. T. Fridovich I. (1993). Iron specificity of the Fur-dependent regulation of the biosynthesis of the manganese-containing superoxide dismutase in *Escherichia coli*. J. Biol. Chem. 268, 5178–5181 8444893

[B52] Radolf J. D. Caimano M. J. Stevenson B. Hu L. T. (2012). Of ticks, mice and men: understanding the dual-host lifestyle of Lyme disease spirochaetes. Nat. Rev. Microbiol. 10, 87–99 10.1038/nrmicro2714 22230951 PMC3313462

[B53] Radolf J. D. Samuels D. S. (eds.). (2010). Borrelia: Molecular Biology, Host Interaction, and Pathogenesis. Norfolk: Caister Academic Press

[B54] Samuels D. S. (2011). Gene regulation in *Borrelia burgdorferi*. Annu. Rev. Microbiol. 65, 479–499 10.1146/annurev.micro.112408.134040 21801026

[B55] Silva F. D. Rezende C. A. Rossi D. C. Esteves E. Dyszy F. H. Schreier S. (2009). Structure and mode of action of microplusin, a copper II-chelating antimicrobial peptide from the cattle tick *Rhipicephalus (Boophilus) microplus*. J. Biol. Chem. 284, 34735–34746 10.1074/jbc.M109.016410 19828445 PMC2787336

[B56] Silva F. D. Rossi D. C. Martinez L. R. Frases S. Fonseca F. L. Campos C. B. (2011). Effects of microplusin, a copper-chelating antimicrobial peptide, against *Cryptococcus neoformans*. FEMS Microbiol. Lett. 324, 64–72 10.1111/j.1574-6968.2011.02386.x 22092765

[B57] Smith A. H. Blevins J. S. Bachlani G. N. Yang X. F. Norgard M. V. (2007). Evidence that RpoS (sigmaS) in *Borrelia burgdorferi* is controlled directly by RpoN (sigma54/sigmaN). J. Bacteriol. 189, 2139–2144 10.1128/JB.01653-06 17158681 PMC1855718

[B58] Sobota J. M. Imlay J. A. (2011). Iron enzyme ribulose-5-phosphate 3-epimerase in *Escherichia coli* is rapidly damaged by hydrogen peroxide but can be protected by manganese. Proc. Natl. Acad. Sci. U.S.A. 108, 5402–5407 10.1073/pnas.1100410108 21402925 PMC3069151

[B59] Srinivasan V. B. Vaidyanathan V. Mondal A. Venkataramaiah M. Rajamohan G. (2012). Functional characterization of a novel Mn2+ dependent protein serine/threonine kinase KpnK, produced by *Klebsiella pneumoniae* strain MGH78578. FEBS Lett. 586, 3778–3786 10.1016/j.febslet.2012.09.007 23010593

[B60] Stadtman E. R. Berlett B. S. Chock P. B. (1990). Manganese-dependent disproportionation of hydrogen peroxide in bicarbonate buffer. Proc. Natl. Acad. Sci. U.S.A. 87, 384–388 10.1073/pnas.87.1.384 2296593 PMC53268

[B61] Stevenson B. Schwan T. G. Rosa P. A. (1995). Temperature-related differential expression of antigens in the Lyme disease spirochete, *Borrelia burgdorferi*. Infect. Immun. 63, 4535–4539 7591099 10.1128/iai.63.11.4535-4539.1995 PMC173648

[B62] Sun D. Lee G. Lee J. H. Kim H. Y. Rhee H. W. Park S. Y. (2010). A metazoan ortholog of SpoT hydrolyzes ppGpp and functions in starvation responses. Nat. Struct. Mol. Biol. 17, 1188–1194 10.1038/nsmb.1906 20818390

[B63] Sy J. (1977). *In vitro* degradation of guanosine 5'-diphosphate, 3'-diphosphate. Proc. Natl. Acad. Sci. U.S.A. 74, 5529–5533 10.1073/pnas.74.12.5529 414222 PMC431794

[B64] Troxell B. Xu H. Yang X. F. (2012). *Borrelia burgdorferi*, a pathogen that lacks iron, encodes manganese-dependent superoxide dismutase essential for resistance to streptonigrin. J. Biol. Chem. 287, 19284–19293 10.1074/jbc.M112.344903 22500025 PMC3365960

[B65] Troxell B. Ye M. Yang Y. Carrasco S. E. Lou Y. Yang X. F. (2013). Manganese and zinc regulate virulence determinants in *Borrelia burgdorferi*. Infect. Immun. 81, 2743–2752 10.1128/IAI.00507-13 23690398 PMC3719580

[B66] Wang P. Dadhwal P. Cheng Z. Zianni M. R. Rikihisa Y. Liang F. T. (2013). *Borrelia burgdorferi* oxidative stress regulator BosR directly represses lipoproteins primarily expressed in the tick during mammalian infection. Mol. Microbiol. 89, 1140–1153 10.1111/mmi.12337 23869590 PMC3772987

[B67] Wang P. Lutton A. Olesik J. Vali H. Li X. (2012). A novel iron- and copper-binding protein in the Lyme disease spirochaete. Mol. Microbiol. 86, 1441–1451 10.1111/mmi.12068 23061404

[B68] Xu Q. McShan K. Liang F. T. (2008a). Essential protective role attributed to the surface lipoproteins of *Borrelia burgdorferi* against innate defences. Mol. Microbiol. 69, 15–29 10.1111/j.1365-2958.2008.06264.x 18452586 PMC2574894

[B69] Xu Q. McShan K. Liang F. T. (2008b). Modification of *Borrelia burgdorferi* to overproduce OspA or VlsE alters its infectious behaviour. Microbiology 154, 3420–3429 10.1099/mic.0.2008/019737-0 18957595

[B70] Xu Q. Seemanapalli S. V. McShan K. Liang F. T. (2006). Constitutive expression of outer surface protein C diminishes the ability of *Borrelia burgdorferi* to evade specific humoral immunity. Infect. Immun. 74, 5177–5184 10.1128/IAI.00713-06 16926410 PMC1594837

[B71] Yang X. Goldberg M. S. Popova T. G. Schoeler G. B. Wikel S. K. Hagman K. E. (2000). Interdependence of environmental factors influencing reciprocal patterns of gene expression in virulent *Borrelia burgdorferi*. Mol. Microbiol. 37, 1470–1479 10.1046/j.1365-2958.2000.02104.x 10998177

[B72] Yang X. F. Alani S. M. Norgard M. V. (2003). The response regulator Rrp2 is essential for the expression of major membrane lipoproteins in *Borrelia burgdorferi*. Proc. Natl. Acad. Sci. U.S.A. 100, 11001–11006 10.1073/pnas.1834315100 12949258 PMC196916

[B73] Yang X. F. Lybecker M. C. Pal U. Alani S. M. Blevins J. Revel A. T. (2005). Analysis of the *ospC* regulatory element controlled by the RpoN-RpoS regulatory pathway in *Borrelia burgdorferi*. J. Bacteriol. 187, 4822–4829 10.1128/JB.187.14.4822-4829.2005 15995197 PMC1169512

[B74] Yui S. Nakatani Y. Mikami M. (2003). Calprotectin (S100A8/S100A9), an inflammatory protein complex from neutrophils with a broad apoptosis-inducing activity. Biol. Pharm. Bull. 26, 753–760 10.1248/bpb.26.753 12808281

